# Pulled-out locking screw re-screwed spontaneously in anterior cervical decompression and fusion with the zero-profile implant system: A case report

**DOI:** 10.1097/MD.0000000000006827

**Published:** 2017-05-05

**Authors:** Yi Yang, Lingli Li, Litai Ma, Junfeng Zeng, Tingkui Wu, Hao Liu

**Affiliations:** Department of Orthopedics, West China Hospital, Sichuan University, Chengdu, Sichuan Province, P. R. China.

**Keywords:** anterior cervical decompression and fusion, complication, locking screw, pull-out, re-screw, Zero-P

## Abstract

**Rationale:**

The zero-profile, standalone device (Zero-P, Synthes GmbH, Switzerland) has been reported to be an effective and safe treatment method with similar clinical outcomes compared with plate. Instrumental complications concerning Zero-P have been little reported. Considering the rarity, we present this amazing case to share with our spinal surgeons and instrument specialists.

**Patient Concerns:**

A 46-year-old man patient presented to our hospital with neck and shoulders pain for 23 years, numbness and weak-ness of right hand for 6 months.

**Diagnoses:**

Hypoesthesia in the right C6 and C7 roots distribution, myodynamia weakness of the right little finger was detected from physical examination. Two-level anterior cervical decompression and fusion (ACDF) using the Zero-P was performed via a classic right Smith–Robinson approach after induction of general anesthesia. Three months postoperative x-rays showed a good position of the implant. Six months postoperative x-rays showed a locking screw at the segment C6/7 pulled out. The patient was diagnosed as screw pullout after ACDF.

**Interventions:**

The patient was treated conservatively with regular follow-up as he was asymptomatic and no evidence of esophageal perforation was detected.

**Outcomes:**

The patient was followed again and 24 months postoperative x-rays also showed the pulled-out locking screw had re-screwed spontaneously. The patient was noticed that a revision surgery was needed if symptoms occur. At present bony union is not reached but he is still asymptomatic.

**Lessons:**

Pulled-out screws re-screwed spontaneously are rare but it does occur. Insertion angle may affect the stability of the Zero-P device, and the repeated micro-motion may be the critical reason of the screw pull-out and re-screwing. The management of screws pull-out after ACDF remains individualized and a revision surgery is not necessary for every patient. Conservative treatment such as orthosis and regular follow-ups may be suitable for some asymptomatic patients.

## 1 Introduction

**Figure 1:**
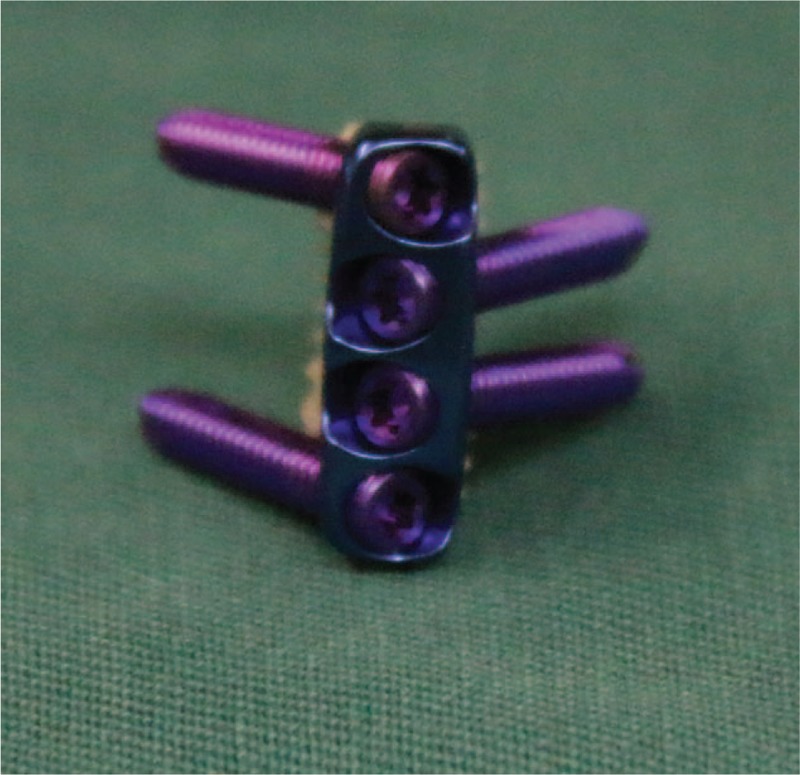
The Zero-P consists of a polyetheretherketone (PEEK) interbody spacer, a titanium alloy plate, and locking head screws.

Anterior cervical decompression and fusion (ACDF) has been regarded as the gold standard for the treatment of degenerative cervical diseases for several decades.^[1]^ Anterior cervical plating has been introduced in several decades in an attempt to increase fusion rates and decrease the incidence of graft extrusion and subsidence; however, anterior cervical plating has also been reported to be associated with some disadvantages and complications such as dysphagia.^[2,3]^ In order to overcome the disadvantages of traditional cervical anterior plating, a new zero-profile device combined an anterior plate with a cage (Zero-P, Synthes GmbH, Switzerland) for ACDF has been developed recently (Fig. 1).^[4–6]^ The Zero-P has been widely used and reported to be an effective and safe treatment method with similar clinical outcomes, lower risk of postoperative dysphagia, shorter operation time, less blood loss, and relatively greater simplicity compared with ACDF with anterior plating for cervical degenerative disc diseases.^[4,7–10]^ Instrumental complications concerning Zero-P have been little reported. We previously reported a case of locking screw pull-out after ACDF using Zero-P, and the patient was treated by conservative method with a followed up of every 6 months as the patient was asymptomatic, the cervical stability was reliable, and no evidence of esophageal perforation was detected.^[11]^ However, after our previous case report has been published and the patient was followed up at the twentieth month, the pulled-out screw was found re-screwed spontaneously. Considering the rarity, we present this amazing case to share with our spinal surgeons and instrument specialists.

## 2 Case report

The patient provided informed consent for the publication of his clinical and radiological data. This study was approved by medical ethical committee of our hospital.

**Figure 2:**
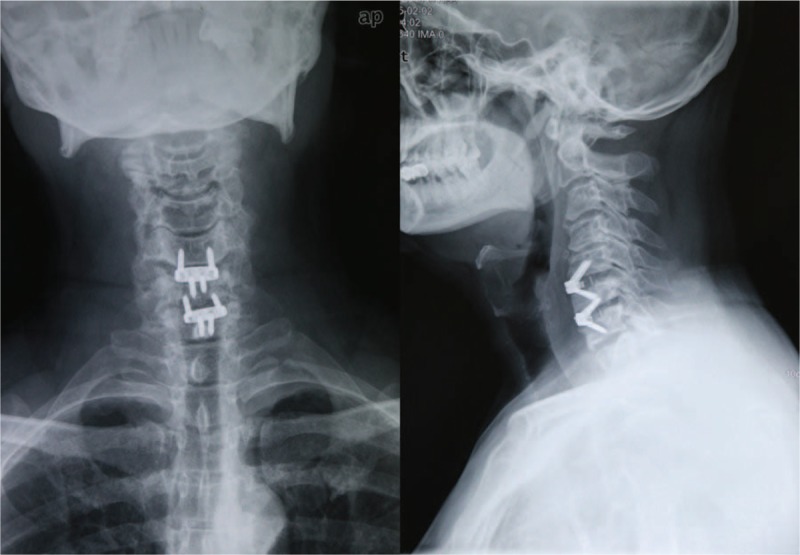
Three months postoperative x-rays showed a good position of the implant.

**Figure 3:**
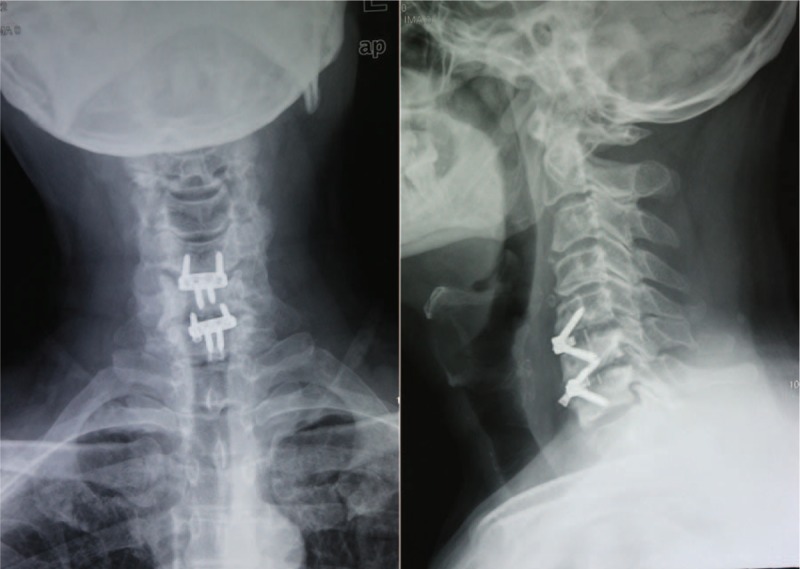
Six months postoperative x-rays showed a locking screw at the segment C6/7 pulled out.

**Figure 4:**
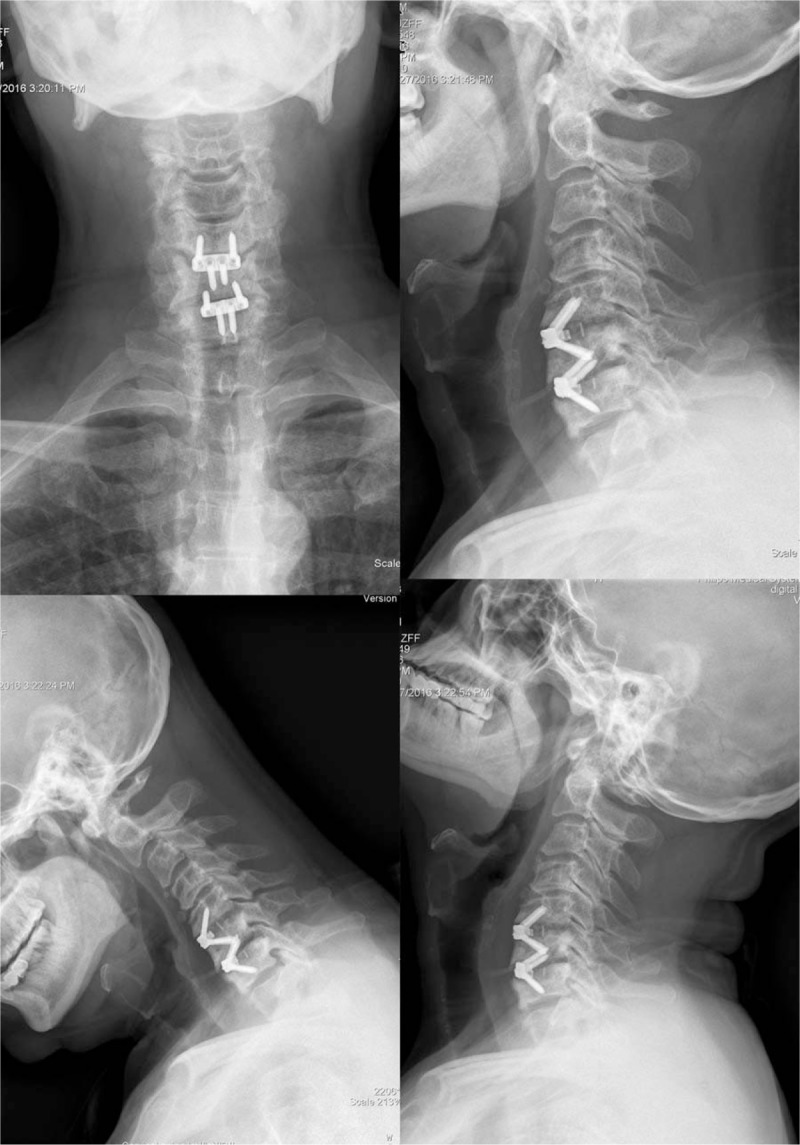
The pulled-out locking screw was found re-screwed spontaneously at the 20th month follow-up.

A 46-year-old male patient presented to our hospital with neck and shoulders pain for 23 years, numbness and weak-ness of right hand for 6 months. Hypoesthesia in the right C6 and C7 roots distribution, myodynamia weakness of the right little finger was detected from physical examination. Radiologic examinations were consisted with his clinical symptoms. ACDF using the Zero-P was performed via a classic right Smith-Robinson approach after induction of general anesthesia. Three months postoperative x-rays showed a good position of the implant (Fig. 2). However, 6 months postoperative x-rays showed a locking screw at the segment C6/7 pulled out (Fig. 3). Regular follow-up was recommended as he was asymptomatic and no evidence of esophageal perforation was detected. To our surprise, the pulled-out locking screw was found re-screwed spontaneously at the 20th month follow-up (Fig. 4). The patient was followed again and 24 months postoperative x-rays also showed the pulled-out locking screw had re-screwed spontaneously. The patient has not reached bony fusion even 24 months after surgery, whether a revision surgery is needed remains controversial. Micro-motion at the segment do existed but the patient was asymptomatic while the cervical stability was reliable. Conservative treatment method with regular followed-up was recommended after discussion with our spinal surgeons in our hospital. The patient was noticed that a revision surgery was needed if symptoms occur. At present bony union is not reached but he is still asymptomatic.

## 3 Discussion

Anterior cervical plate has been introduced to ACDF in 1980s, complications of unlocked plates were not low, especially the plates and screws loosening, and even screws pullout.^[12]^ Locking screws were reported to achieve better stability and decrease the instrumental complications.^[13,14]^ Locking screws pull-out after ACDF is not a common complication which can cause serious secondary complications such as esophageal perforation, screw missing, pharyngoesophageal diverticulum perforation, and wound infections. Instrumental complications concerning zero-profile implants have been little reported. Hofstetter et al^[15]^ reported a case of migration of blade after application of a zero-profile anchored spacer in a retrospective cohort study. Barbagallo et al^[8]^ reported a case of screw displacement after application of Zero-P synthes in a retrospective case series study.

We previously reported a case of locking screw pull-out after ACDF using Zero-P synthes.^[11]^ The patient was asymptomatic and no evidence of esophageal perforation was detected after examinations of esophagoscopy and barium meal examination. The patient was obeyed to avoid excessive neck movement and followed regularly. The pulled-out locking screw was found re-screwed spontaneously at the 20th month follow-up and the patient was treated conservatively with regular follow-up. To the best of our knowledge, this is the first report of locking screw re-screwed spontaneously after pullout in ACDF using Zero-P synthes.

A lot of factors can affect the screws pullout strength such as basic screw designs (screw size, conical screws or not, thread type, expandable screws), insertion techniques (such as pilot hole size, pretapped hole or self-tapping, insertion angle, bicortical fixation), bone quality of the patient, bony fusion or not, use of orthosis, and so on.^[16]^ The screw pullout of this patient may be attributed to the following reasons: repeated micro-motion because of non-fusion of the segments drove the screw out; uncomplaisance of the patient: too early and excessive neck activity especially excessive extension activity; possible intraoperative inopportune insertion angle caused “false locking” and decreased the stability of the locking screws. Re-screwing spontaneously after pullout seems unbelievable and the mechanism remains unclear. In our opinion, 2 possible explanations may be applied: (1) the bone absorption at the segment decreased the pull-out strength and screwing force, the pulled-out screw re-screwed under the force of oesophageal compression; (2) repeated micro-motion at the segment drove the pulled-out screw into the titanium alloy plate slowly and coincidently. However, this is just our hypothesis and we have no evidence to certify it.

The management of screws pull-out after ACDF remains individualized. In deed a revision surgery is not necessary for every patient. Several factors should be considered: patient's symptom such as dysphagia; level of the screws pull-out; conditions of bony fusion; possibility of esophageal perforation; cervical stability; results of esophagoscopy, cervical x-rays, and barium meal examination. If the patient is asymptomatic, and no evidence of esophageal perforation is detected, conservative treatment such as orthosis and regular follow-ups is recommended. Of course a revision surgery is needed for some patients to avoid severe complications such as esophageal perforation and esophageal fistula. Pulled-out screws re-screwed spontaneously are rare but it does occur. Conservative treatment with regular follow-up may be suitable for some patients.
